# Supplementary material for: Biseugenol Exhibited Anti-Inflammatory and Anti-Asthmatic Effects in an Asthma Mouse Model of Mixed-Granulocytic Asthma
Source: Molecules. 2020 Nov 18;25(22):5384. doi: 10.3390/molecules25225384 (PMC7698799; doi:10.3390/molecules25225384)
Supplement: Supplementary file 1 [file molecules-25-05384-s001.pdf]

Biseugenol Exhibited Anti-Inflammatory and Anti-Asthmatic Effects in An Asthma Mouse Model of Mixed-Granulocytic Asthma

Vitor Ponci [1], Rafael C. Silva [2], Fernanda Paula R. Santana [2], Simone S. Grecco [3], Célia Regina M. Fortunato [3], Maria A. Oliveira [4], Wothan Tavares-de-Lima [4], Clarice R. Olivo [5], Iolanda de Fátima L. Calvo Tibério [5], Kaio S. Gomes [6], Carla M. Prado [7,*] and João Henrique G. Lago [6,*]

1 Departament of Chemistry, Universidade Federal de São Paulo, São Paulo 09972-270, Brazil; vitor.ponci@gmail.com

2 Departament of Biological Sciences, Universidade Federal de São Paulo, São Paulo, 09972-270, Brazil; rafael.cossi.silva@gmail.com (R.C.S.); fe.paula.roncon@gmail.com (F.P.R.S.)

3 Departament of Biotechnology and Health Innovation, Universidade Anhanguera, São Paulo, 05145-200, Brazil; grecco.simone@gmail.com (S.S.G.); celiarmfort@gmail.com (C.R.M.F.)

4 Departament of Pharmacology, Institute of Biomedical Sciences, Universidade de São Paulo, São Paulo, 05508-000, Brazil; cidora@yahoo.com (M.A.O.); wtavares@usp.br (W.T.-d.-L.)

5 School of Medicine, Universidade de São Paulo, São Paulo, 01246-000, Brazil; clariceolivo@gmail.com (C.R.O.); iocalvo@uol.com.br (I.d.F.L.C.T.)

6 Human and Natural Science Center, Universidade Federal do ABC, São Paulo, 09210-580, Brazil; kaiosouza.quimica06@gmail.com

7 Departament of Bioscience, Universidade Federal de São Paulo, São Paulo, 11015-020, Brazil

* Correspondence: carla.prado@unifesp.br (C.M.P.); joao.lago@ufabc.edu.br (J.H.G.L.)

SUPLEMENTARY MATERIAL

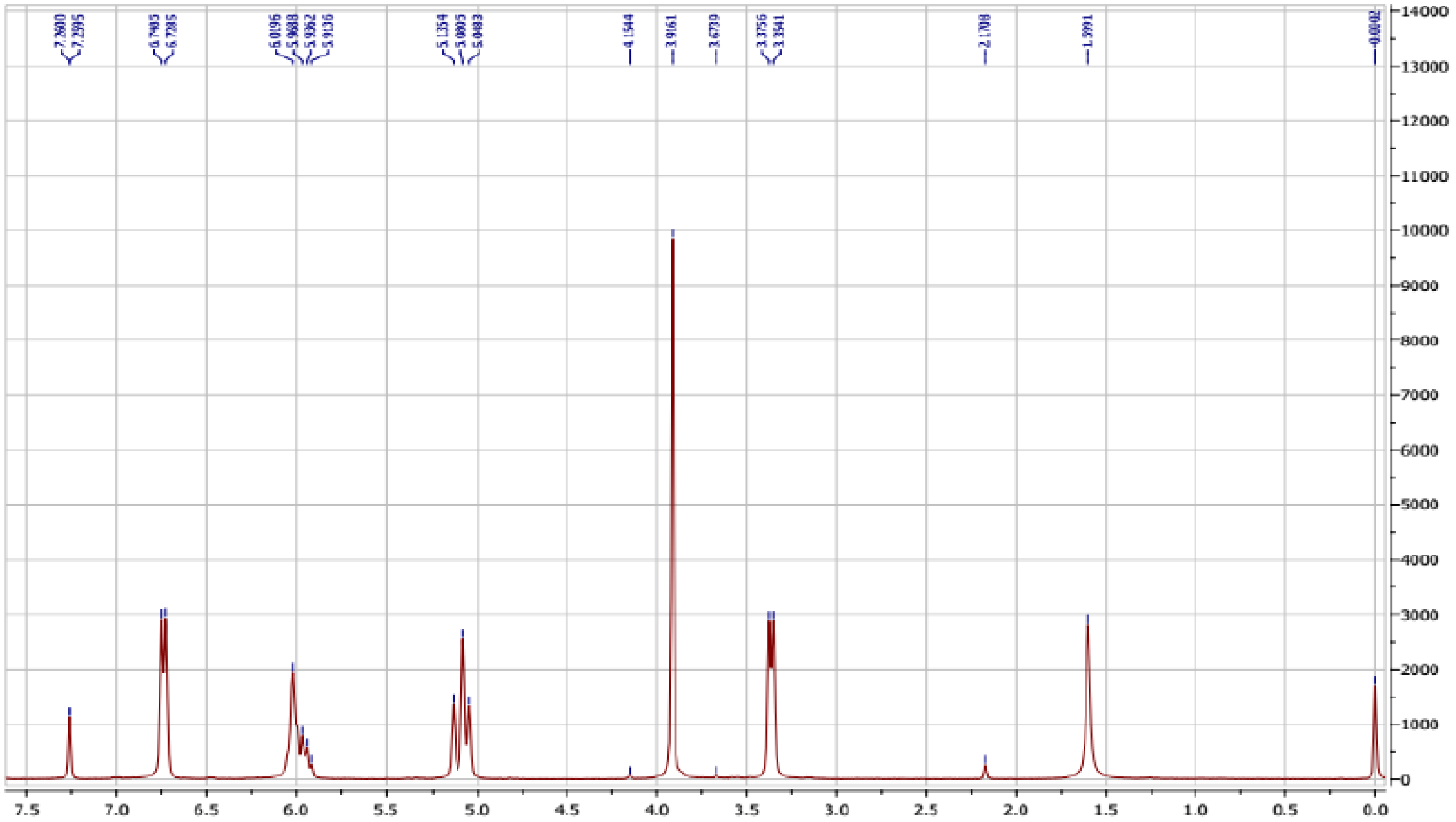

Figure S1. ^{1}H NMR spectrum of biseugenol (δ, $CDCl_3$, 300 MHz)

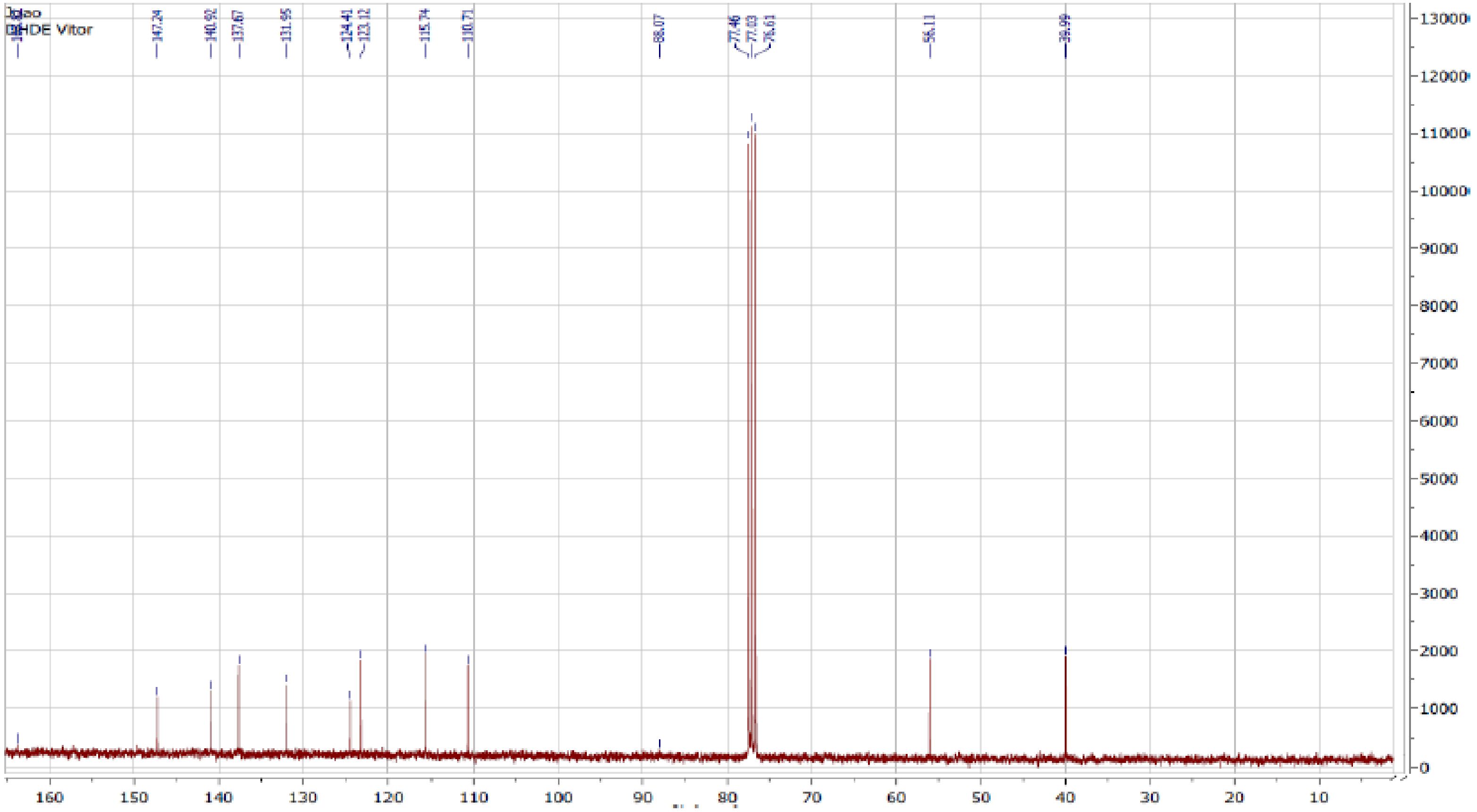

Figure S2. ^{13}C NMR spectrum of biseugenol (δ, $CDCl_3$, 75 MHz)

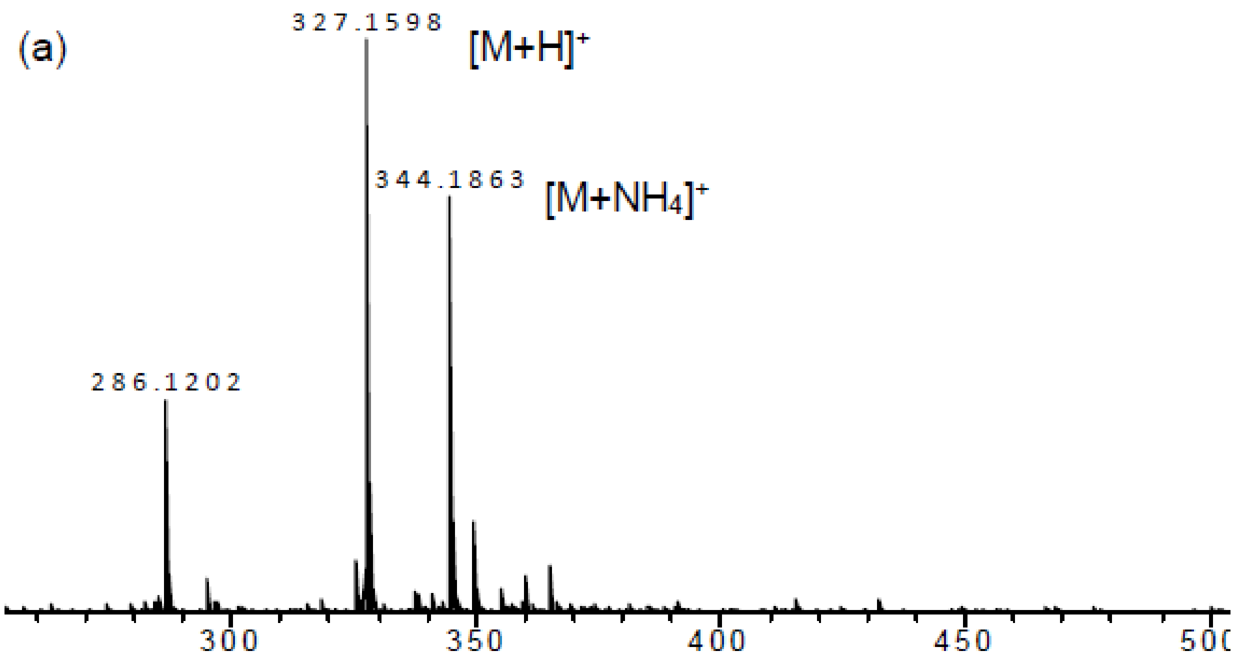

Figure S3. HRESIMS (positive mode) of biseugenol
